# Packets-to-Prediction: An Unobtrusive Mechanism for Identifying Coarse-Grained Sleep Patterns with WiFi MAC Layer Traffic

**DOI:** 10.3390/s23146631

**Published:** 2023-07-24

**Authors:** Dheryta Jaisinghani, Nishtha Phutela

**Affiliations:** 1 Department of Computer Science, College of Humanities, Arts, and Sciences, University of Northern Iowa, Cedar Falls, IA 50613, USA; 2 Department of Computer Science and Engineering, BML Munjal University, Gurugram 122413, India; nishtha.phutela@bmu.edu.in

**Keywords:** WiFi, sniffer, MAC layer, sleep detection, machine learning

## Abstract

A good night’s sleep is of the utmost importance for the seamless execution of our cognitive capabilities. Unfortunately, the research shows that one-third of the US adult population is severely sleep deprived. With college students as our focused group, we devised a contactless, unobtrusive mechanism to detect sleep patterns, which, contrary to existing sensor-based solutions, does not require the subject to put on any sensors on the body or buy expensive sleep sensing equipment. We named this mechanism Packets-to-Predictions(P2P) because we leverage the WiFi MAC layer traffic collected in the home and university environments to predict “sleep” and “awake” periods. We first manually established that extracting such patterns is feasible, and then, we trained various machine learning models to identify these patterns automatically. We trained six machine learning models—K nearest neighbors, logistic regression, random forest classifier, support vector classifier, gradient boosting classifier, and multilayer perceptron. K nearest neighbors gave the best performance with 87% train accuracy and 83% test accuracy.

## 1. Introduction

Sleep deprivation affects all our physiological and psychological functions, resulting in a disturbed circadian rhythm of the body and, thus, weakening the immune response, which ultimately affects our vital organs [1,2]. Even though each individual has different sleep requirements, various expert agencies such as the American Academy of Sleep Medicine (AASM), Sleep Research Society (SRS), and the National Sleep Foundation (NSF) recommend 7–9 hour of sleep daily. Various surveys conducted by the Centers for Disease Control and Prevention (CDC) and Sleep Foundation found that 74.6% of high school students and 32.5% of adults do not sleep for adequate hours, and overall, one-third of US adults sleep less than 7 hour per night [3,4].

Although sleep disorders affect all sections of the human population, we specifically focused on college students in this work. The primary reason is that the young and vibrant youth drive the country’s future [5,6,7]. A lack of qualitative and quantitative sleep severely impacts the academic performance of college or university students. Several literary works have shown a strong correlation between good grades and a good night’s sleep for college students [8,9,10,11,12]. Students score better grades if they sleep well, attend classes regularly, and discuss class topics with their peers and instructors. Insufficient sleep impairs their cognitive capabilities, which reduces their attention and concentration span in the classroom [13]. Bad grades affect students long-term in terms of a student’s perseverance, confidence, diligence, and self-discipline, which decide the professional success of an individual [14,15,16].

We survey the efforts conducted as of today in the direction of detecting sleep quality and quantity. Currently, the solutions available to monitor sleep are costly specialized devices requiring special training to operate. For example, polysomnography is the *Gold* standard for sleep monitoring in healthcare [17]. Polysomnography uses an electroencephalogram, electrooculogram, electromyogram, electrocardiogram, and pulse oximetry to generate a polysomnogram (PSG) that helps study sleep patterns in detail [18]. Although polysomnography provides an in-depth analysis of a person’s sleep quality, it cannot be used long-term due to the discomfort caused by the obtrusive use of sensors. Recently, the research world of mobile computing came up with wearable body sensors such as accelerometers and heart-rate sensors [19,20,21]. A subject has to wear these sensors while s/he sleeps. Generally, the human population finds sleeping uncomfortable while wearing these special sensing devices on their body [22,23].

In this work, we propose to develop *P2P—From Packets To Prediction*—an unobtrusive system to monitor the quality of night sleep among college students that does not require them to wear any sensors on the body. Given that college students are avid smartphone users, we propose to rely on passively collected WiFi traffic from a student’s smartphone. Our major hypothesis is that the students who experience symptoms of disturbed sleep tend to use their smartphones whenever they wake up at night. Most commonly, smartphones are connected over WiFi to obtain network access; thus, they transmit WiFi traffic whenever used. We believe that the WiFi medium access control (MAC) layer traffic comprises signatures showing a strong correlation between periods of activity during awake hours and inactivity during sleeping hours. WiFi MAC layer traffic is layer two network traffic, as per the protocols defined in IEEE 802.11 standard [24], that is not affected by the physical orientation of a person or device. On the contrary, several recent works in the wireless domain used physical layer (or layer 1) channel characteristics such as channel state information (CSI), received signal strength indicator (RSSI), or backscatter technology to identify sleep patterns. These approaches require special equipment such as a special tag, sensor, or software-defined radio device [25,26,27]. The use of specialized equipment limits the scalability of these approaches and adds to the deployment costs. Another approach to developing an unobtrusive sleep sensing system is relying on WiFi traffic that can be collected passively with WiFi infrastructure or sniffers. The former approach requires a professional and large-scale setup of production WiFi controllers such as from Cisco or Aruba [28]. The limitation of this approach is the cost and effort of deployment. Most home setups and small-scale academic institutions do not have such a setup. The latter option is to rely on passive sniffing by WiFi access points or low-cost sniffing devices. The intuition behind this approach is that the WiFi MAC layer traffic collected by the passive sniffing carries unique signatures based on smartphone usage that can help us detect different sleep patterns. With current off-the-shelf access points having inbuilt sensors and available sniffing devices, deployment cost and effort is not a problem with this approach.

**The research question** that we attempt to answer in this work is—*Do our smartphones emit sufficient and necessary WiFi MAC layer traffic that can help us identify coarse-grained windows of “sleep” and “awake” periods.* The premise of this work is that students who have disturbed sleep patterns use smartphones connected over WiFi for network access. To establish that, we conducted surveys with 53 undergraduate students to understand their sleep patterns. Specifically, our questionnaires revealed the quality and the quantity of a student’s sleep and the number of times students woke up to check their smartphones. These surveys confirm that students use their smartphones when they wake up at night. Going forward, we collected four nights of the controlled dataset where a subject woke up every hour to manually study the signatures that helped us prove the hypothesis that “sleep” and “awake” periods exist in the WiFi MAC layer traffic. Given that manual signature identification is not a scalable solution, we collected four more nights of the controlled WiFi MAC layer traffic, which accounts for ≈56 hour worth of data in two different WiFi environments—home and student dormitories. We used this data to train six machine learning models to help us automate the “sleep” and “awake” period detection for both the home and university environments. We present the student survey results, data collection methodology, WiFi MAC layer traffic overview, manual pattern identification, and background on machine learning models and metrics in Section 3. Our analysis revealed that out of the six models we trained, the K nearest neighbors model performed best with 87% train accuracy and 83% test accuracy. We present the training process and accuracy of the models in Section 4.

We identify the major contributions of this paper as follows:

1. We conducted surveys with students to establish that students with disturbed sleep patterns use smartphones when they wake up at night.
2. We presented the design of P2P, which is a passive sniffing mechanism to collect WiFi MAC Layer traffic when a subject is sleeping.
3. We presented the manual identification process of “sleep” and “awake” periods from WiFi MAC layer traffic.
4. We trained six machine learning models to automate the “sleep” and “awake” period process from WiFi MAC layer traffic.

We present the related work in Section 2, a discussion on limitations and the use-case in Section 5, and conclude with future research directions in Section 6.

## 2. Related Work

This section discusses the current state-of-the-art approaches for detecting various sleep-related events.

**Smartphone based approaches**: In these approaches, authors develop smartphone apps to track the sleep cycles of the subjects. For example, the authors in [29] have studied depression in college students using smartphones and wearable sensors. The sleep features such as sleep time, wake-up time, and sleep duration were inferred using passive sensing from mobile phones. These authors hypothesize that students who are depressed might have more variation in their sleep time and wake-up time. The sleep inferences were drawn using four mobile phone sensors—ambient light, audio amplitude, activity, and screen on/off. They also used a sleep classifier. Likewise, the authors in [30] developed a system called *Sleep Hunter* for Android phones to detect sleep stages: wake, light sleep, and REM. Sleep Hunter is a system that uses the smartphone sensor’s microphone, accelerometer, and light sensor to collect data related to environmental disturbances, such as light and noise, and events during sleep, such as movements, coughing, and snoring, to detect three sleep stages (e.g., REM, deep, and light sleep). These authors use specific feature-extraction methods for each sleep-related event identified above based on their physical characteristics. The authors in [31] also conducted a similar work using smartphone sensors to infer a subject’s sleep quality. The differentiating factor in their work is the inferred sleep quality with physical environment sensing—for example, light, phone usage, stationary or mobile, and sound level in the room. Researchers have also tried to rely entirely on raw acoustic signals collected from the smartphone’s microphone to decipher sleep events. One such work is presented in  [32], where authors developed an Android app to collect acoustics data and train decision-tree-based algorithms for sleep classification.

**WiFi based approaches**: In these approaches, the authors rely on WiFi signals. The authors in [33] developed a system *Serene* to capture respiration rate and body motions with the help of WiFi channel state information (CSI). CSI-based methods are dependent on the location of the WiFi transmitter and receiver. The authors analyze the impact of respiration on the multipath components of WiFi signals to quantify the effect of small breathing movements on the CSI signals. They evaluate their approach with sleep monitoring as a case study. “Wi-Sleep” [34] investigates the feasibility and accuracy of using WiFi signals to monitor sleep patterns. “Wi-Sleep” utilizes the changes in WiFi signal patterns caused by human movement during sleep. The system employed signal processing techniques and machine learning algorithms to extract sleep-related information from the WiFi signals. The Wi-Sleep system achieved high accuracy in detecting sleep onset, sleep stages (such as REM and non-REM sleep) and sleep quality metrics (including sleep efficiency and awakenings). It requires external tx-rx pairs for collecting the radio signals. The authors in [35] present *SMARS*, a sleep monitoring system via ambient radio signals collected with external embedded devices. SMARS exploits commodity ambient radio signals to recognize sleep stages and assess sleep quality. SMARS works in a non-obtrusive manner without any body contact. The subject needs to set up one single link between two commodity radios. The tx-rx pair has multiple antennas in its implementation. Similar to these works, the authors in [27] also leverage the channel state information to develop a fusion and signal processing method. Their proposed approach requires channel state information from multiple antennas, and it then extracts accurate respiration and body movement information. Their deep learning algorithm can classify four stages of sleep monitoring. Finally, the work presented in  [28] is close to what we have proposed in this article. The authors developed a system that relies on production WiFi controllers in a large-scale deployment to collect RTLS data. Since a person carries more than one WiFi device these days, they collect data from all the WiFi devices a person owns and classify their sleep.

Although all these works are commendable, our aim is slightly different in this paper. We want to refrain from app-based solutions because past research shows that it is difficult to motivate people to install and use apps. We do not want to mandate the subject to interact with the phone in any artificial manner. We rely on the subject’s natural behavior of waking up and using the phone. We do not want to deploy external sensors/servers/controllers as it adds to deployment cost and effort. We highlight the differences between WiFi-based approaches and P2P in Table 1. Our approach in P2P is simply recording and analyzing WiFi MAC layer traffic to classify “sleep” and “awake” patterns for a given subject. We leverage WiFi MAC layer traffic because of the following reasons—(1) all WiFi devices transmit frames that comprise the MAC layer traffic, in accordance to IEEE 802.11 standard [24]; (2) all WiFi devices follow similar algorithms for scanning WiFi networks, associating with an access point, data transmission, and connection maintenance; (3) MAC layer traffic is independent of a person or device’s orientation; and (4) Passive sniffers can easily record MAC layer traffic, eliminating the need for specialized network hardware. We present the WiFi MAC layer traffic overview in Section 3.2 and Section 3.3.

## 3. Materials and Methods

In this section, we first discuss the results of surveys that we conducted with college students. Then, we explain the system overview of P2P, the data collection procedure, and provide an overview of WiFi MAC Layer traffic. Next, we explain how WiFi MAC layer traffic carries signatures that help us infer “sleep” and “awake” periods. Finally, we explain machine learning algorithms that we trained our models with, features used, and performance metrics.

### 3.1. Motivation Survey

We conducted an internal survey with 53 college students to understand their sleep patterns better. Specifically, the objective of this survey was to know:

1. How many hours of night sleep are students getting?
2. How do they rate their sleep quality?
3. How often do they wake up at night to use the smartphone and then go back to sleep?

For the first two questions, students answer the questions on a 10–point Likert scale ranging from 0 to 10, with 0 being the lowest. The data were then divided into three categories: less than six, between six and eight, and greater than eight. Depending upon the question, the meaning of their response changes. For example, for (1), the number on the scale denotes hours while for (2), the number on the scale denotes the quality, with 0 being the lowest and 10 being the highest. For the third question, we ask them the number of times they wake up for their phone use. We give them options—0, 1, 2, 3, 4, 5, 6, and >6.

We also asked the students about their smartphone usage. It turns out that 71% of them use smartphones before sleeping. The use of smartphones before falling asleep also results in disturbed sleep patterns, which is the focus of this paper.

**Figure 1:**
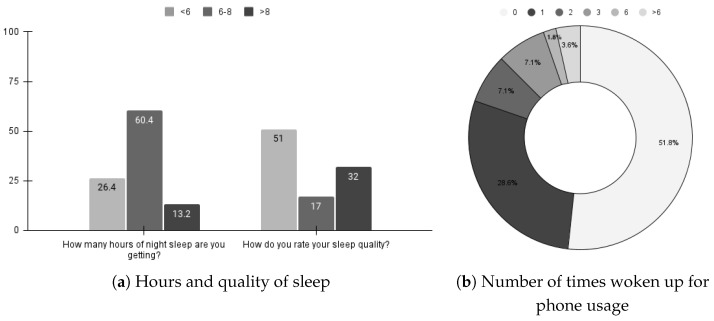
Results of internal student sleep survey.

We present the survey results in Figure 1. Figure 1a shows the results for Questions 1 and 2. We found that only 13.2% of the students could sleep for more than 8 hours, 60% of students slept between 6–8 hours, and 26.4% of the students slept for <6 hours. We also note that 51% of students report poor sleep quality. That means they experience disturbed sleep. So we tried to assess if they use phones when they wake up while sleeping.

We present those results in Figure 1b. We report the percentage of students who wake up to use the phone and then return to sleep. While we see that 51.8% of students do not wake up for phone usage, the remaining 48.2% of students do wake up at least once to use their phones, which is enormous. It gives us strong evidence that smartphone usage at night adds to their disturbed sleep. It also motivates us to leverage WiFi traffic for detecting students’ sleep patterns.

### 3.2. Data Collection with P2P

**Figure 2:**
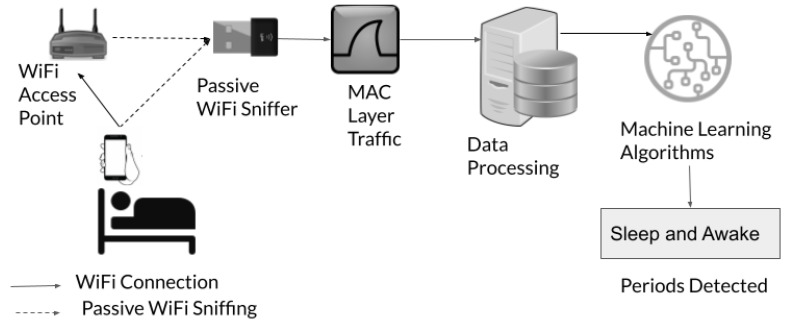
Data Collection Setup.

**System Overview**: With the P2P system setup, the data are collected, as shown in Figure 2. A subject sleeps with a smartphone that is connected to a WiFi access point for network connectivity. The model of the access point is Optronix RL821GWV, which operates in 2.4 GHz and supports IEEE 802.11a/b/g/n. We use a USB WiFi adapter, TP-Link TL-WN722N, that supports IEEE 802.11n as the passive sniffer with the help of monitor mode configuration. It is connected to a Lenovo Ideapad 130 Core i5 8th Gen—(8 GB RAM/1 TB HDD/Ubuntu 20.04.3 LTS) with Intel Core i5-8250U CPU. We capture the MAC layer traffic with command line utility tshark, version 3.2.3.

To maximize the likelihood of recording all the MAC layer traffic destined for the access point and transmitted by the access point, we place a sniffer nearby the access point. The sniffer passively listens to the same frequency channel on which the WiFi access point operates. The sniffer does not inject any active traffic into the channel. Once the data collection is finished for the night, the MAC layer traffic captured by the sniffer is processed for the manual inspection of signatures and training machine learning algorithms.

We detect patterns for two periods in the traffic collected—“awake” and “sleep”. Study participants help us collect the initial dataset. We define a *subject* as the person who is undergoing disturbed sleep and s/he wakes up multiple times at night, uses the phone, and goes back to sleep again. The study participants are healthy young adults sleeping in comfortable environments and bedding facilities. The study participants mimic the disturbed sleep pattern by waking up every hour, using the phone for a few minutes, and returning to sleep. For normal sleep patterns, they sleep through the night without using their phones. All the timestamps when the participant woke up and when the participant went back to sleep are noted manually and serve as ground truth for our dataset. The study participant alternates between “Awake” and “Sleep” periods every hour. We recruited three study participants but each night has data for exactly one study participant. The duration of data collection is at least 6 hours per night with a maximum of 8 hours per night.

We collected eight nights of data in home and university environments. Out of this, we used four nights of data collected in the home environment for manually demonstrating the correlation between the type and quantity of WiFi traffic and a subject’s sleep quality with the help of manual pattern identification. Then, we considered the entire eight nights of data for training machine learning algorithms to predict “sleep” and “awake” periods.

In order to evaluate the efficacy of the developed approach, P2P, across phone models, we used different phones for the data collection. Table 2 shows the details of these phones.

**Figure 3:**
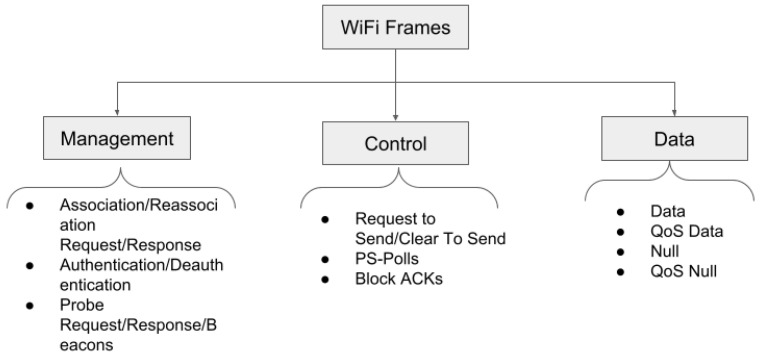
Types of WiFi Frames.

**WiFi MAC Layer Traffic Overview**: At the MAC layer, WiFi clients communicate by exchanging or broadcasting three types of frames—management, control, and data frames. Figure 3 shows an abstract overview of the types of WiFi frames. the management frames help manage the network by allowing clients to discover, associate, and authenticate with access points or access points to broadcast their presence. The control frames enable controlled access to the medium; for example, they help clients and access points to decide who should transmit next. Finally, the data frames are the frames that carry data. tshark or Wireshark is a utility is to capture these frames as WiFi packets. Thus, frames and packets are used interchangeably in this paper.

A closed analysis of these frames reveals that they carry various signatures that help us decipher various physical activities [36]. Below, we list some of these signatures:

1. WiFi clients send a burst of probe requests when their screens are turned on after a few seconds (generally, 10–15 seconds).
2. WiFi clients send keep-alive frames to the access points, such as null data frames, when their screens are off for a few hours. This is performed to keep the connection on with the access point.
3. WiFi clients frequently send data frames (QoS or non-QoS) when they are in active use.

We use these signatures to decipher periods of “sleep” and “awake” during a subject’s night sleep time. We explain the process in detail in the next section.

### 3.3. Manual Pattern Identification

This section presents our technique to identify “sleep” and “awake” periods from WiFi traffic. As mentioned in the previous section, WiFi traffic has signatures that help us decipher a subject’s activities. In this work’s context, we focused on finding the signatures that can identify a subject’s sleeping vs. awake stage.

The WiFi communication protocol requires WiFi devices, such as smartphones, to scan WiFi networks before establishing a connection with an access point. Once a connection is established, the data can be exchanged between a WiFi device and the associated access point. We denote this as the active mode. If this connection is broken, the device again has to undergo scanning and connection establishment, adding to an unnecessary communication delay. Therefore, WiFi devices and access points have connection keep-alive protocols that ensure an active connection is not torn down completely when the WiFi device is not in active use. Most commonly, for battery-saving purposes, WiFi devices have to be in power-save mode when they are not being used; for example, a person turns the screen off of a smartphone for a long time, and that is when connection keep-live protocols are triggered.

This paper’s unobtrusive sleep pattern identification mechanism builds up on this phenomenon. When sleeping, a subject’s phone’s screen is turned off; thus, it gradually enters into a power-save mode, and on waking up, the subject turns the phone’s screen on, which transitions the phone’s WiFi card to active mode. Based on the current mode of operation, the type of WiFi frame and frequency of transmission of that WiFi frame changes. For example, smartphones transmit QoS null or null data frames in power-save mode. When transitioning to active mode, a smartphone can probe access points to announce that it is now exiting power-save mode, and it transmits probe request frames. When the smartphone transitions to active mode, it has ongoing data communication with the access point, transmitting data or QoS data frames.

**Figure 4:**
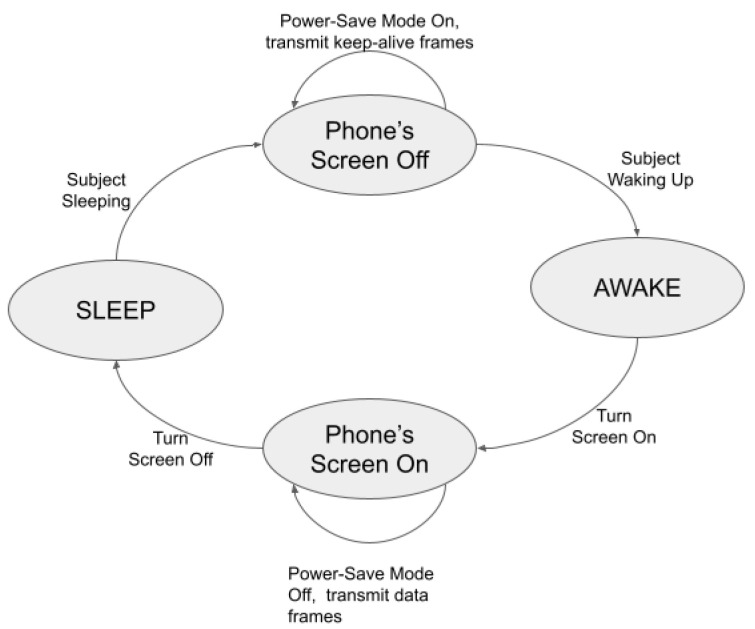
Sleep and awake transition, along with the phone’s state.

We demonstrate this process in Figure 4. When the subject is in a sleep state, the phone’s screen is off but transmits keep-alive frames. When the subject transitions to the awake state, they turn on their phone’s screen, which is when the phone transmits discovery frames and data frames. During these transitions, two types of frame-related events are possible:

- Event 1: Change in Frame Type
- Event 2: Change in Quantity of a Frame Type

We would like to point out here that tracking these events does not hamper the privacy of the subject under consideration because—(1) the WiFi MAC layer traffic is already encrypted, and (2) P2P does not need to know the content of any frame. To understand these two events better, we analyzed four nights of data collected by subjects. For two of these four nights, the subject had a regular, undisturbed night sleep, and for the remaining two nights, the subject had a disturbed sleep where the subject woke up every hour, used the phone for a few minutes, and then went back to sleep.

**Figure 5:**
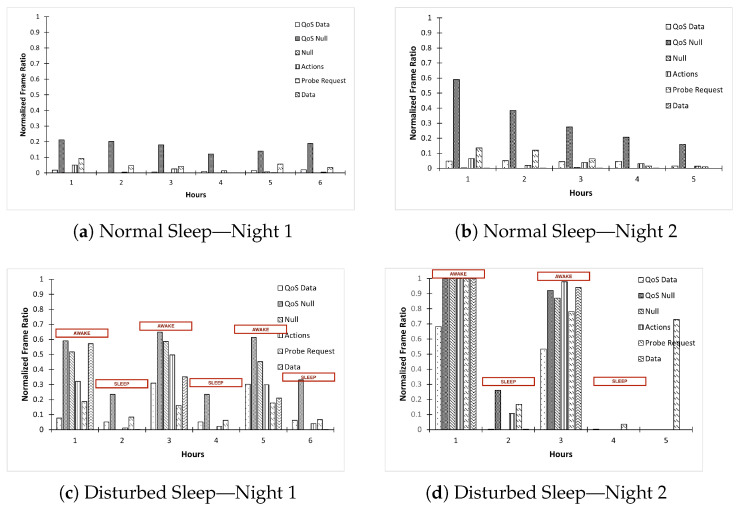
Manual “Sleep” and “Awake” period identification for normal and disturbed sleep for two nights.

We present the results of this data collection in Figure 5. The number of frames on the Y-axis, represented as *normalized frame ratio*, are normalized to a scale of 0 to 1 using min–max normalization to compare the quantity across different nights. The X-axis denotes the hour of sleeping or awake. Notice that in Figure 5a,b, when the subject is sleeping through the night, in the initial one hour, the QoS data frames are being sent, demonstrating that the subject was using the phone before sleeping. Gradually, as the subject sleeps and does not turn on the phone again throughout the night, both Event 1 and Event 2 occur. For Event 1, frame types change. Now the phone is transmitting only keep-alive frames. So, we see QoS null frames, null frames, probe request frames, and action frames. For Event 2, the frequency and, hence, the quantity of these frames also severely reduce, signifying that the phone is not involved in any active data transfer, and with every hour passing, the quantity keeps reducing, signifying that the phone’s WiFi is transitioning to a deep sleep state.

Likewise, as shown in Figure 5c,d, we notice significant changes in Event 1 and Event 2 when the subject is undergoing a disturbed sleep, keeps waking up, and uses the phone every alternate hour. Whenever the subject transitions from the sleep to awake stage, one of the following happens—QoS data frames increase, probe requests increase, and action frames increase. In contrast, when the subject transitions from the Awake to Sleep stage, either the quantity of these frames is significantly reduced or these frames disappear altogether.

Although we can establish a correlation between the type/quantity of frame types and sleep/awake state, it is hard to quantify any thresholds for the number of frames or patterns for types of frames manually in a universal way. Thus, in the next section, we train various machine learning models to improve the proposed approach’s versatility and compare their accuracy.

### 3.4. Machine Learning Approaches

**Dataset and Algorithms**: We considered four nights of data from the home environment and four nights from the university environment to train our machine learning models. Each environment had two nights of normal sleep and two nights of disturbed sleep data. We used multiple combinations of nights to train our models. We trained six machine learning algorithms, namely—K nearest neighbors (KNN), logistic regression (LR), random forest classifier (RFC), support vector classifier (SVC), gradient boost (GB), and multilayer perceptron (MLP). MLP is a sequential model with two dense layers. The first dense layer has the same number of neurons as the number of features/events in the input data frame. The second dense layer has a single neuron with a sigmoid activation function, which is commonly used for binary classification tasks. The activation function used is the rectified linear unit (ReLU).

**ML Features**: The features used for these machine learning algorithms are motivated by the events we discussed in Section 3.3. Recall that Event 1 denotes a change in frame type, and Event 2 denotes a change in quantity of a frame type. Thus, we use the following features for these algorithms—(a) timestamp of a frame’s transmission, (b) type of a frame, and (c) inter-frame arrival time. The timestamp of a frame’s transmission is the epoch time recorded in tshark for that frame, the type of a frame is the type defined in IEEE 802.11 standard, and inter-frame arrival time is the time elapsed between recording two frames of the same type. It is critical to note that when the inter-frame arrival time for a frame type is low, it signifies that it is being transmitted frequently (hence, more quantity). Likewise, if the inter-frame arrival time is high, it signifies a reduced transmission frequency for that frame type (hence, lesser quantity).

**Performance Metrics**: We measured the performance of these models with the following metrics—accuracy, precision, recall, and F1-score. For a disturbed night’s sleep, the data contain sleep and awake periods. Our machine-learning algorithms aim to predict these two periods correctly. A correctly predicted sleep period (CSP—correct sleep period) is a true positive, and a correctly predicted awake (CAP—correct awake Period) period is a true negative. When a sleep period is classified as an awake period (SAP—sleep identified as awake period), it is denoted as a false negative, and when an awake period is predicted as a sleep period (ASP—awake identified as sleep period ), it is denoted as a false positive.

- The accuracy of the models is defined as the total number of correctly predicted sleep and awake periods out of the total number of sleep and awake periods. The accuracy is reported in percentage.
  (1) $$\begin{array}{c} accuracy=\frac{\left(\#CSP+\#CAP\right)}{\#CSP+\#CAP+\#SAP+\#ASP} \end{array}$$
- Precision is defined as—of all the total predicted sleep periods, how many are correct sleep periods? Precision is measured on a scale of 0 to 1.
  (2) $$\begin{array}{c} precision=\frac{\#CSP}{\#CSP+\#ASP} \end{array}$$
- Recall is defined as—of all the total actual sleep periods, how many are correctly identified sleep periods? Recall is measured on a scale of 0 to 1.
  (3) $$\begin{array}{c} recall=\frac{\#CSP}{\#CSP+\#SAP} \end{array}$$
- F1-score is the weighted mean of Precision and Recall. F1-score is measured on a scale of 0 to 1.
  (4) $$\begin{array}{c} F1-Score=\frac{2*precision*recall}{precision+recall} \end{array}$$

In order to help students, the system should not tell them that they are sleeping fine when they are not. That means the system should identify disturbed sleep correctly. The machine learning terminology indicates that we should aim to minimize false positives, i.e., an awake period identified as a sleep period and, thus, should have high precision. Conversely, sleep periods incorrectly identified as awake would mean a low recall (or high false negatives). Ideally, we expect a high recall for accurate prediction; however, a low recall does not have severe after-effects on students.

## 4. Results

**Figure 6:**
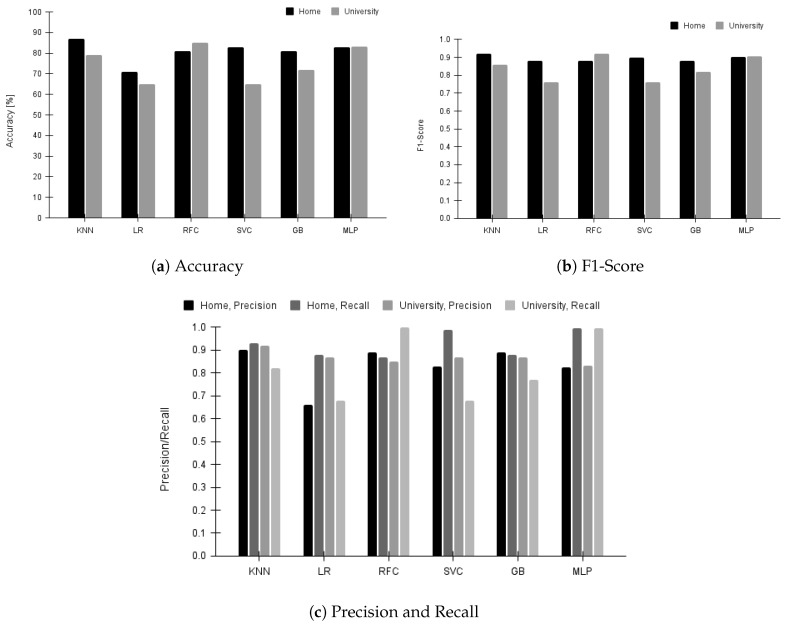
Training performance of machine learning models for all nights in the home and university environment.

**Figure 7:**
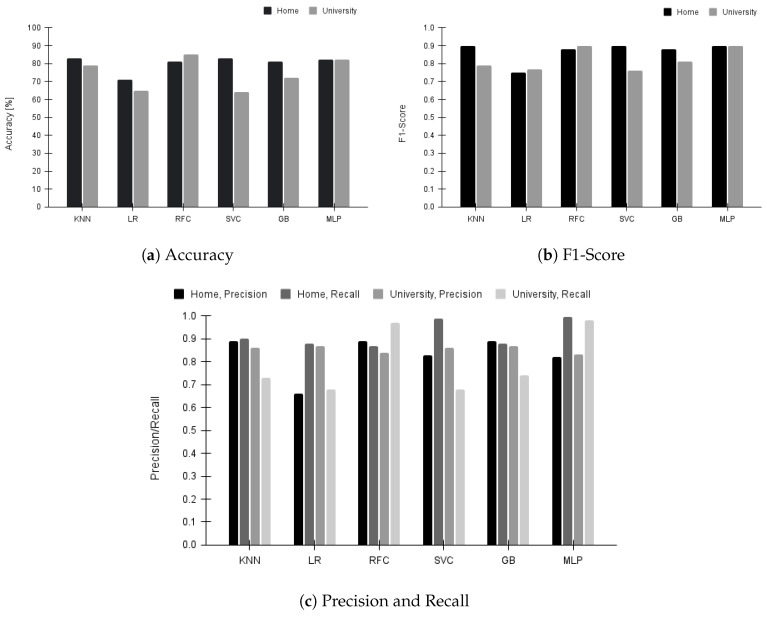
Testing performance of machine learning models for all nights in the home and university environment.

All our machine learning models use a 70–30% split for training and testing, respectively. The computational complexity of the machine learning models follows the standard upper bounds with specific training times for each model as follows—KNN: 0.24 seconds, LR: 0.74 seconds, RFC: 11.1 seconds, SVC: 11 minutes, GB: 4.82 seconds, and MLP: 83.21 seconds. We trained the models on Google CoLab with GPU: RTX 3050 4 GB and RAM: 16 GB [37]. We report the training results of each model in Figure 6 and the testing results from each model in Figure 7. All the results are presented for both home and university environments. The accuracy results are summarized in Figure 6a for training performance. We noticed that KNN performs best with 87% accuracy in the home environment. From Figure 6b, we observe that while all models give decent F1-scores in both environments, MLP gives the best overall prediction. We report precision and recall in Figure 6c. Across all models in both environments, KNN gives the best precision, which aligns with our expectation that this system should not wrongly predict awake windows as sleep. We notice that even recall is high enough across models, with KNN providing the best prediction. This indicates fewer chances of predicting a sleep window as an awake window. Along with KNN, MLP also provides high recall, but its precision is not as good as KNN. We observe that the SVC and LR models exhibited poor performances with our datasets. Although these models’ accuracy is more than 70%, their precision and recall are the lowest. The main factor contributing to the under-performance of these models is the presence of a class imbalance in the dataset—sleep periods are more than awake periods. As shown in Figure 7, we do not see any significant deviations in the performance results on the testing dataset, which conforms that our models are trained correctly.

Although this work aims to identify awake windows to discover if a subject is having disturbed night sleep, the system, when used in the real world, should not bother its subjects by wrongly identifying normal sleep as disturbed sleep. To verify this, we used a dataset where the subject sleeps through the night and fed that to all the trained algorithms. In this case, KNN and CNN identified sleep windows correctly with a high precision of 1, and none of the algorithms identify any awake windows. To conclude, we find KNN performs best in both environments. However, as a supervised machine learning algorithm, it becomes tedious to annotate the dataset for supervised training as the dataset grows. Hence, we propose to continue to develop sound neural networks such as CNNs for detecting sleep and awake patterns for massive datasets spanning several nights.

We notice a slight drop in the performance of our models when we migrate from home to a university environment. For example, the accuracy of KNN drops from 87% to 79%; its F1-score drops from 0.9 to 0.8, and precision and recall drop from 0.89 –> 0.86 and 0.9 –> 0.73, respectively. The reason behind these kinds of drops is the environmental characteristics. The home environment is comparatively more silent than the university environment concerning WiFi traffic because the number of WiFi devices in the home is way less than the number of WiFi devices in the dormitories of a university. Fewer devices emit less traffic, so the sniffer’s fidelity is better at home than in the university environment. The quality of the captured traffic directly impacts the performance of all machine learning models.

**Figure 8:**
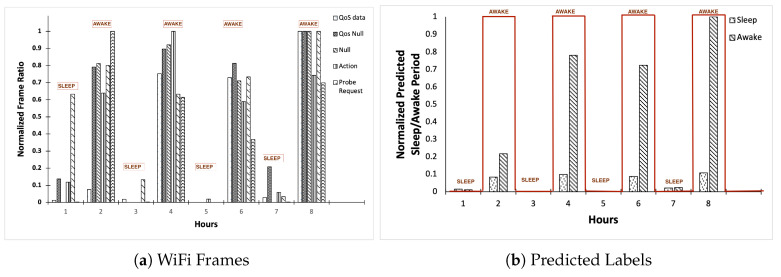
ML model prediction of the sleep and awake periods in the real world.

Finally, we now demonstrate using P2P for predicting sleep and awake periods by showing a correlation between WiFi frames and the predicted machine learning labels. We show the results in Figure 8. Figure 8a shows the variation in the type and quantity of WiFi frames with manual identification of sleep and awake periods, and Figure 8b shows the predicted labels for these periods every hour. We performed a majority vote to make the final prediction from per-hour predicted labels. It can be seen that Hours 1, 3, and 5 are identified as sleep periods and Hours 2, 4, 6, and 8 are identified as awake periods manually with frames in Figure 8a. Each hour matches the sleep and awake periods with the machine learning predictions in Figure 8b.

Given the versatility of WiFi MAC layer traffic across WiFi devices, these trained models are generic and can be used for different devices in different environments.

## 5. Limitations and Discussion

This section discusses some limitations and a use-case of this work.

**Limitations**: First, this is not a real-time solution; we have to collect data and then process it offline, so there is always an associated delay. However, we believe that sleep data should be analyzed over several weeks to give meaningful feedback to the subject. Second, currently, P2P is tested for one subject per MAC Layer Traffic capture. P2P will work with multiple subjects per traffic capture file; however, its evaluation and validation are not yet completed. Sniffer fidelity must also be tested with multiple subjects per traffic capture file. Third, unlike radio signals, for example, CSI-based approaches that can categorize stages of sleep, this technique cannot identify the stages of sleep, such as deep or REM. Although this is a limitation, the idea of this work is not to analyze the stages of sleep; we want to know if students are experiencing disturbed sleep patterns and help them reduce their phone usage at night. Thus, both these limitations do not limit the broader applicability of this work. Fourth, in its current version, P2P is not trained for deep neural networks, and thus, its accuracy is highly dependent on the feature engineering described in this paper. As the scale of the data increases, P2P’s efficacy can be considerably enhanced with deep neural networks. Finally, we evaluated P2P in a highly controlled setting where the study participants woke up every alternate hour to imitate disturbed sleep patterns. In the real world, P2P should be tested for true disturbed sleep patterns where the subject does not undergo a predetermined sleep pattern.

**Use-case**: Even though we identify a few limitations of this work, we foresee an impactful application for P2P. We discussed in the introduction that if the students sleep better, they are more attentive in class, receive better grades, and become better professionals when they graduate. Most students undergo disturbed sleep patterns without realizing the after-effects of the lack of sleep. If officials in the universities and families in the homes adopt P2P, then they obtain coarse-grained predictions on “how” the student is sleeping through the night. The identification of disturbed sleep patterns proactively gives an opportunity for the caretakers to mend the sleeping schedules and habits of the students. The students can be motivated to improve their quality of sleep significantly before more sophisticated medical intervention is involved. The ease of deployment and cost-effectiveness make P2P an attractive solution to implement in dormitories and homes.

## 6. Conclusions and Future Work

We were able to develop a simple system, P2P, to identify “sleep” and “awake” periods by analyzing WiFi MAC layer traffic. The advantage of the proposed approach is that it does not need any modifications to a subject’s usual lifestyle, i.e., no app installations, no new radios to be installed, no changes to the access points needed, and no special sensors to be worn. It is a cost-effective technique that does not need any special deployments. Universities and homes can quickly adopt this to detect disturbed student sleep patterns. We are now working on—(1) extending P2P’s evaluation for more real-world scenarios where a subject’s sleep pattern is unknown and there is more than one subject per traffic capture file; (2) training the models for deep neural networks to improve the scalability and applicability of P2P; and (3) finally, developing techniques with which we can build feedback mechanisms about the sleep quality for the students, such as HCI-based solutions to help them sleep better.

## Figures and Tables

**Table 1:** Comparing P2P with Existing WiFi-based Solutions.

Title	Sensing Modality	Environmental Dependence	Specialized Equipment	Sleep Stage
Serene [33]	WiFi Multipath and CSI	Yes	Yes	Sleep–Awake Markers with Body Movements
Wi-Sleep [34]	WiFi CSI	Yes	Yes	Sleep–Awake Markers with Body Movements
SMARS [35]	WiFi CSI	Yes	Yes	Sleep–Awake–REM–NREM
SleepMore [28]	WiFi RTLS	No	Yes	Sleep–Awake Markers
P2P (this work)	WiFi MAC	No	No	Sleep–Awake Markers

**Table 2:** Details of phones used for data collection.

Phone Model	OS	WiFi Capability
Realme X7 Max	Android v12	802.11ax (2.4 GHz|5 GHz)
Oppo Reno 7 pro	Android v12	802.11 a/b/g/n/ac/6 (2.4 GHz|5 GHz)
OnePlus 8	Android v12	Wi-Fi 802.11 a/b/g/n/ac/6 (2.4 GHz|5 GHz)

## Data Availability

The data presented in this study are openly available here—https://github.com/Nishtha13/Packet-to-Prediction.git, accessed on 19 July 2023.
